# Identification of chronic wasting disease prions in decaying tongue tissues from exhumed white-tailed deer

**DOI:** 10.1128/msphere.00272-23

**Published:** 2023-10-06

**Authors:** Paulina Soto, Francisca Bravo-Risi, Rebeca Benavente, Stuart Lichtenberg, Mitch Lockwood, J. Hunter Reed, Rodrigo Morales

**Affiliations:** 1 Department of Neurology, The University of Texas Health Science Center at Houston, Houston, Texas, USA; 2 Centro Integrativo de Biologia y Quimica Aplicada (CIBQA), Universidad Bernardo O’Higgins, Santiago, Chile; 3 Department of Veterinary and Biomedical Sciences, Minnesota Prion Research and Outreach Center, University of Minnesota, Saint Paul, Minnesota, USA; 4 Texas Parks and Wildlife Department, Kerrville, Texas, USA; Colorado State University, Fort Collins, Colorado, USA

**Keywords:** prions, chronic wasting disease, protein misfolding cyclic amplification (PMCA), real-time quaking induced conversion (RT-QuIC), white-tailed deer, exhumation

## Abstract

Chronic wasting disease (CWD) prions cause fatal neuropathies in farmed and free-ranging cervids. The deposition of prions in natural and humanmade environmental components has been implicated as a major mechanism mediating CWD spread in wild and captive populations. Prions can be deposited in the environment through excreta, tissues, and carcasses from pre-clinical and clinical animals. Furthermore, burial of CWD-positive animals may reduce but not completely mitigate prion spread from carcasses into the surrounding environment. Here, we analyzed exhumed, decaying deer carcasses for the presence of CWD prions. By analyzing tongue tissues through the protein misfolding cyclic amplification (PMCA) technique, we were able to identify seven out of 95 exhumed white-tailed deer carcasses as CWD prions carriers. Confirmatory analyses were performed using the real-time quaking-induced conversion (RT-QuIC) technique. In addition, we evaluated the potential contamination of the pens that housed these animals by swabbing feeders and waterers. PMCA analyses of swabs confirmed CWD contamination on farming equipment. This work demonstrates the usefulness of PMCA to detect CWD prions in a variety of contexts, including exhumed/decaying tissues. In addition, this is the first report demonstrating swabbing coupled with PMCA as a method for the detection of prion seeding activity on naturally exposed surfaces. Considering that this study was focused on a single site, further studies should confirm whether prion amplification assays are useful to identify CWD prions not only in animals but also in the environment that contains them.

**IMPORTANCE:**

Environmental contamination is thought to be a major player in the spread of chronic wasting disease (CWD), a fatal prion disease affecting a wide variety of cervid species. At present, there are no officially approved methods allowing for the detection of prion infectivity in environmental components. Importantly, animal as well as anthropogenic activities are thought to contribute to prion environmental contamination. Here, we detected CWD prions in exhumed white-tailed deer carcasses by using the protein misfolding cyclic amplification (PMCA) assay. In addition, we identified CWD prions in feeders used within the infected facility. These results highlight the potential role of PMCA in identifying prion infectivity in a variety of scenarios, ranging from decaying tissues to farming equipment.

## OBSERVATION

Chronic wasting disease is a fatal neurodegenerative disease affecting farmed and free-ranging cervids (1). CWD is caused by exposure to infectious prions (PrP^Sc^), misfolded proteins that self-replicate by templating their conformational features into the non-infectious version of the host cellular prion protein (PrP^C^) (2 – 4). Prions are very resistant to degradation and can persist in the environment for years while maintaining their infectious properties. Experimentally, it has been shown that murine-adapted bovine prions (301V strain) spiked in buried cattle heads remain infectious for several years (5). Another report demonstrated that naïve deer are readily infected when placed in pens that housed CWD-infected animals several years prior (6). Importantly, CWD transmission was demonstrated by the inoculation of naïve mule deer with infected carcasses (6). These studies and many others highlight the threat that CWD carcasses pose to the environment, potentially creating exposure risks for both wild and farmed animals.

Some of the current disposal options for carcasses from CWD-positive, suspected, or exposed animals include (i) cremation (900°F for 4 h) or (ii) treatment with sodium hydroxide and heat (300°F at up to 70 psi). Although these treatments are expected to eliminate CWD infectivity, the remaining ashes are recommended to be buried deeply to avoid potential prion spills due to the remarkable recalcitrance of prions even at extreme temperatures. A third (iii) option is to bury the decaying corpses or tissues on-site or at an authorized landfill without pre-treatments (https://www.aphis.usda.gov/animal_health/animal_diseases/cwd/downloads/cwd-program-standards.pdf), with the assumption that prions sequestered deeply in soil environments will be inaccessible to susceptible animals. All these practices are designed to contain prion infectivity and restrict its access to naïve cervids, other animal species, and the environment. Unfortunately, considering previous evidence on the persistence and resistance of infectious prions to environmental conditions, the burial of carcasses may inadvertently generate unwanted foci of CWD infectivity. In fact, multiple studies demonstrate that soils avidly bind infectious prions (7) and even increase infectivity titers (8). Worrisomely, it was demonstrated that prion infectivity contained in buried cattle heads migrates gradually, in a lateral and transversal manner, into soils as a result of rainfalls (5). On the contrary, other studies suggest that prions concentrate on the soil surface with minimal mobility due to their uptake by plants that are subsequently consumed by ruminants (9 – 11). The evidence mentioned above indicates that although the burial of untreated prion-infected carcasses helps in containing prion infectivity, it may also create a potential reservoir of this particular infectious agent.

**Fig 1:**
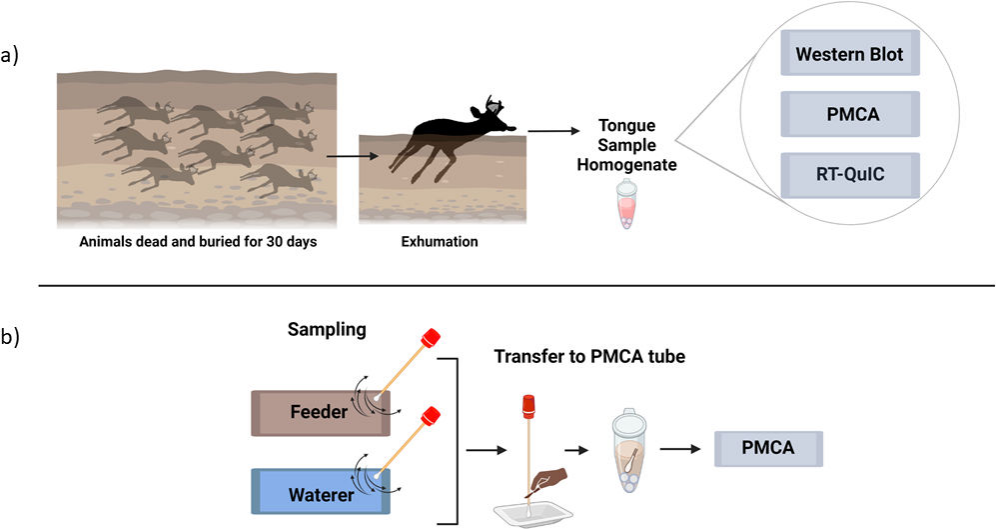
Schematic representation of sample collection and analyses. (a) Ninety-five white-tailed deer carcasses were exhumed 30 days after burial and tongue specimens were collected. These samples were shipped to UTHealth-Houston for further analyses including western blotting, PMCA, and RT-QuIC. (b) Feeders and waterers in contact with deer were swabbed and tested by PMCA. The full description of these procedures can be found in the Supplementary Information linked to this article. Figure created with BioRender.com.

**Fig 2:**
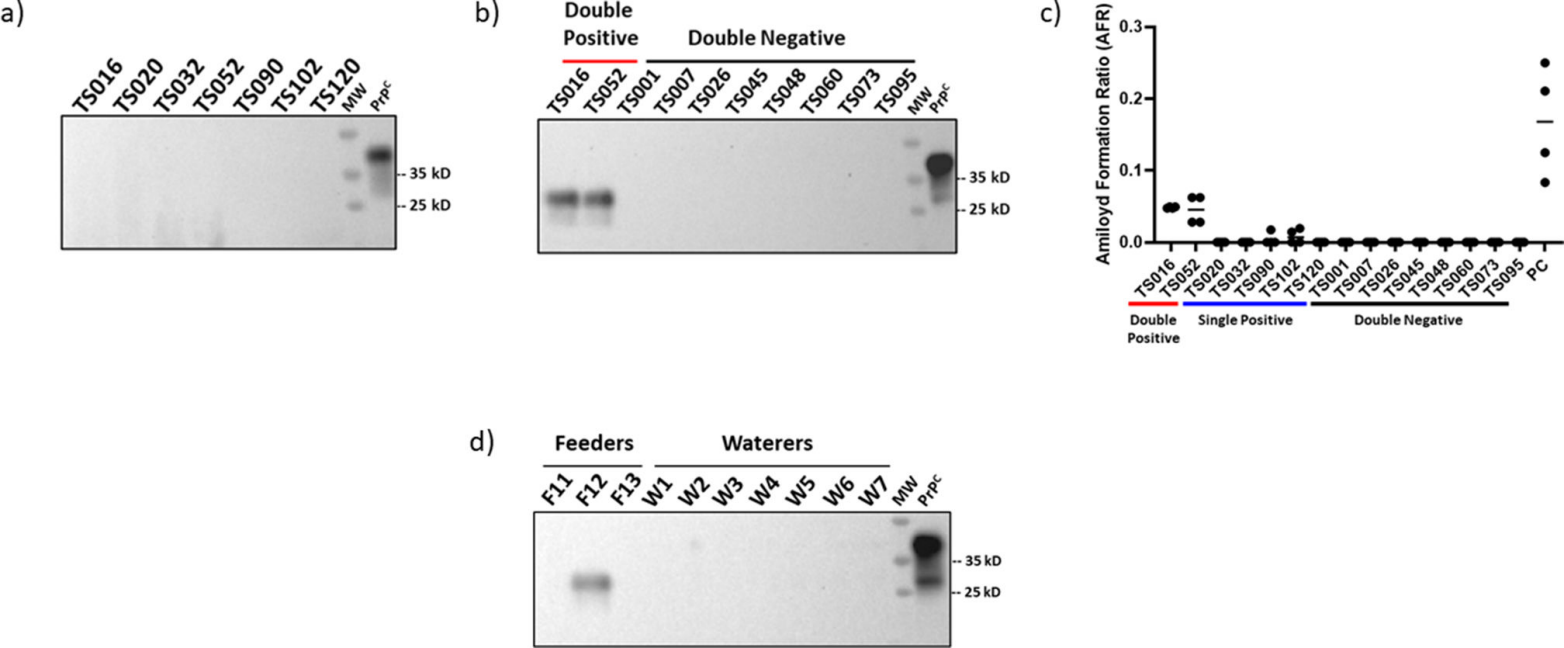
Representative results of western blot, PMCA, and RT-QuIC screening on exhumed tongue samples. (a) Representative samples displaying negative PrP^Sc^-associated signals in western blot after proteinase K treatment. (b) PMCA analyses in exhumed tongue tissues. Results in this panel correspond to a fourth PMCA round, and they depict eight samples displaying negative signals in both replicates (“Double −”) and the two samples that were positive for PMCA in both replicates (“Double +”). The PMCA procedure was performed as described in Ref.^13^ with no modifications besides the use of tongue tissue. Codes at the top of panels (a) and (b) represent codes from individual animals. (c) Representative RT-QuIC data including all PMCA-positive samples and eight representative samples that provided PMCA-negative results in both replicates. “PC” represents RT-QuIC data of a retropharyngeal lymph node from a pre-clinical white-tailed deer used as a positive control. (d) PMCA analysis of representative feeders (F11, F12, F13) and waterers (W1–W7) swabs. As in (b), these results also correspond to a fourth PMCA round. Numbers at the right of panels (a), (b), and (d) correspond to molecular weight (MW) markers. All samples in (a), (b), and (d) were treated with proteinase K with the exception of “PrP^C^” which corresponds to the brain extract of tg1536 mice (used to prepare PMCA substrate) that is utilized as an electrophoretic mobility and antibody specificity control. All blots were developed using the 8H4 antibody.

CWD is officially diagnosed in animal tissues by ELISA or immunohistochemical (IHC) means. Unfortunately, these techniques have relatively low sensitivity and are incompatible with environmental samples (12). The protein misfolded cyclic amplification (PMCA) technique is a powerful method able to detect low levels of CWD prions (13) in a wide range of environmental (11, 14, 15) and biological samples (16 – 20). In this study, we identified CWD prions in exhumed tissues (tongues) from decaying white-tailed deer carcasses using the PMCA technique. Tongue tissues were obtained from farmed, CWD-suspected white-tailed deer carcasses that were euthanized, buried for approximately 30 days, and exhumed (Fig. 1a). Considering the decaying stage of carcasses, tongues were collected for this study as they presented good integrity compared to other tissues. The selection of this tissue is also supported by previous research demonstrating that (i) lesions in this tissue facilitate prion infection (21) and (ii) PMCA is able to detect CWD prions in tongues (22). Tongue samples from 95 animals were collected for this study by Texas Parks and Wildlife Department (TPWD) personnel. These samples were then shipped to UTHealth-Houston facilities for further PMCA analyses. First, we dissected the tongues and collected the apical area, which included muscles and papillae. Then, we directly analyzed tissue homogenates (10% wt/vol) for their content of proteinase K-resistant PrP^Sc^ by western blot as described in the Supplementary Information. This analysis did not provide signals associated with PrP^Sc^ (Fig. 2a), preliminarily suggesting that samples contained either low levels of CWD prions or were devoid of them.

Next, we tested the same samples using PMCA (13). All samples were analyzed in duplicate by two investigators who were blinded to the identity of the samples. PMCA results were interpreted after four rounds, and samples were considered positive if at least one of the replicates resulted in positive signals. Among the 95 samples tested, seven showed the presence of PrP^Sc^ (Fig. 2b). PMCA-positive samples were distributed among two that were positive in both replicates (Fig. 2b), and five provided positive signals in a single replicate (not shown). We believe that positive PMCA signals in a single replicate for these five samples can be explained in the possible limited amounts of prions that will not be present (or present in quantities below the threshold of detection) in some of the aliquots. Similar outcomes have been observed in other experiments using blood (19, 23) and in the multiple replicates needed in RT-QuIC analyses (18). All 88 remaining samples were PMCA-negative in both replicates. To confirm these results, we analyzed the seeding activity of all samples using the RT-QuIC technique. Importantly, our analyses confirmed our previous findings, as the double-positive tongue samples provided positive RT-QuIC signals in all replicates, while the double-negative samples did not display seeding activity (Fig. 2c). For the five specimens that provided a single-positive result in PMCA, no RT-QuIC seeding activity was identified in any of the four replicates for three of these samples. The remaining two samples displayed seeding activity in some replicates but did not reach the threshold of 3/4 positive replicates to be classified as CWD-positive. Considering these results, all these five samples were considered as CWD-negative when using the RT-QuIC assay.

Next, we evaluated the plausible scenario that the CWD-infected deer contaminated their housing premises when alive. We swabbed feeders (*n* = 13) and waterers (*n* = 12) from several pens used to contain these animals (Fig. 1b). Swab pieces were directly placed in tubes containing PMCA substrate and immediately submitted to the prion amplification assay (see Supplementary Information). Although swabs from all waterers provided negative signals after four PMCA rounds, a single feeder swab provided a clear positive signal, suggesting the presence of seeding-competent CWD prions (Fig. 2d).

One of the main limitations hindering the proper containment of the CWD epidemic is the lack of sensitive diagnostic methods. Although the currently USDA-approved methods (ELISA and IHC) have been proven to be good *postmortem* diagnostic techniques, their relatively low sensitivity coupled with the minute levels of prions in accessible samples from pre-clinical animals illustrate the current limitations in this area. An additional limitation is that these techniques are not practical for detecting prions in environmental components. The latter is critical, as convincing evidence suggests that environmental prion contamination is involved in a significant proportion of all CWD cases (24). Fortunately, the invention and further refinement of seeding amplification assays such as the PMCA and RT-QuIC assays provide hope in addressing the previously mentioned limitations. Here, we used PMCA to detect the potential presence of CWD prions in exhumed white-tailed deer carcasses that were at advanced stages of decomposition. Using tongue tissues, we demonstrated the presence of prions in the retrieved carcasses. Our results demonstrate that PMCA is able to detect prions in decaying tissues, confirming this technique as a good tool for identifying infectious prions in a variety of field-relevant situations. It is relevant to mention that signals were evaluated after four PMCA rounds, suggesting that decaying tongue tissues contained low infectivity titers. Along this line, we cannot discard that the decaying tongue tissue matrix interferes with the PMCA reaction, delaying the detection of CWD prions. Unfortunately, control (CWD-free) decaying tongue tissue from deer was not available to us, and for that reason, experiments to measure the extent of this potential inhibition were not attempted. Considering the high efficiency and differential performance of different sample types, PMCA results can only be estimated as positive or negative. For that reason, bioassays should be ideal for estimating the infectivity titers present in the PMCA-positive tongue tissues described in this study. Future experiments in our laboratory will address this relevant question.

Importantly, our results were partially confirmed by RT-QuIC. From our firsthand experience, RT-QuIC works with similar sensitivities compared with PMCA when prion-contaminated brain tissues are used. However, RT-QuIC and PMCA performance significantly vary depending on the sample type (Morales et al., unpublished). In this study, we were also able to track infectivity at housing premises (i.e., at feeder stations) by simple swabbing, demonstrating for the first time the use of PMCA in the screening of CWD prions in naturally infected farming equipment.

Unfortunately, the CWD status (i.e., early-preclinical, late-preclinical, and clinical) of the animals at the moment of euthanasia was unknown, and this did not allow us to make associations between the disease stage and prion detection. Another limitation of this study is that the results presented here corresponded to a single site, limiting the validity of our results to this specific location (e.g., specific prion strains present on this site, PrP polymorphic variation in this cohort of animals, composition of materials used in feeders and waterers, etc.). Nevertheless, we believe that the findings described in this study warrant future research testing additional sites and potentially CWD-contaminated premises to validate the use of PMCA as an animal and environmental diagnostic tool.

## SUPPLEMENTAL MATERIAL

Supplemental text msphere.00272-23-s0001.docx Supplemental Materials and Methods.

ASM does not own the copyrights to Supplemental Material that may be linked to, or accessed through, an article. The authors have granted ASM a non-exclusive, world-wide license to publish the Supplemental Material files. Please contact the corresponding author directly for reuse.
