# Supplementary material for: Identification of chronic wasting disease prions in decaying tongue tissues from exhumed white-tailed deer
Source: mSphere. 2023 Oct 6;8(5):e00272-23. doi: 10.1128/msphere.00272-23 (PMC10597447; doi:10.1128/msphere.00272-23)
Supplement: Supplemental text — Supplemental Materials and Methods. [file msphere.00272-23-s0001.docx]

**MATERIALS AND METHODS**

**Tongue samples.** Tongue specimens from 95 previously buried and partially decaying white-tailed deer carcasses were collected for this study by Texas Parks and Wildlife Department (TPWD) personnel. Deer carcasses were covered with dirt and piled on top of each other. Tongues from these carcasses were retrieved after being buried for approximately 30 days. After collection, tongue tissues were shipped to UTHealth-Houston facilities for protein western blot, misfolding cyclic amplification (PMCA) and real/time quaking-induced conversion (RT-QuIC) analyses. First, all the samples were homogenized at 20% weight/volume (w/v) in phosphate buffer saline (PBS, Hyclone PBS, GE Healthcare Life Sciences) supplemented with a protease inhibitors cocktail (cOmplete™, EDTA-free Protease Inhibitor Cocktail, Roche). The tissue used in this study included tongues’ apices. The homogenized samples included muscle tissue and papillae. Tongue samples were considered hard tissues. For that reason, these were homogenized in 2mL CK-28-R (hard tissue) tubes coupled to a Precellys® 24 homogenizer. For homogenization, we used program number 5 as often as necessary until tissues were fully homogenized. Samples were placed on ice between homogenization cycles. All resulting homogenates were stored at -20 °C until use.

**Swabbing.** Swabs were commercially acquired (Fisherbrand™ Swab/Tube Applicators). Prior to swabbing, swab tips’ were submerged in sterile PBS (Hyclone PBS, GE Healthcare Life Sciences). Each tested surface (waterers and feeders) was swabbed five times in horizontal and vertical directions. Swabs were placed in clean conical tubes and maintained at room temperature until transferred to UTHealth-Houston facilities. At UTHealth-Houston, samples were stored at -20 °C until used.

**PMCA substrate.** Substrate for PMCA was prepared as previously described (1) using brains of tg1536 mice (2). All animal procedures were covered by protocol number AWC-22-0039. Tg1536^+/+^ (homozygous) mice were humanly euthanized by CO_2_ inhalation following protocols approved by the UTHealth-Houston’s Animal Welfare Committee. Mice’s brains were collected after cardiac perfusion with ice cold PBS (Hyclone PBS, GE Healthcare Life Sciences) supplemented with 5 mM EDTA (Promega). Perfused brains were snap-frozen in liquid nitrogen and stored at -80 °C until used. Brains were homogenized at a concentration of 10% w/v in PMCA conversion buffer (PBS (Hyclone PBS, GE Healthcare Life Sciences), supplemented with 1% Triton X-100 (Sigma) and 150 mM NaCl (Sigma-Aldrich) and a protease inhibitor cocktail (cOmplete™, Protease Inhibitor Cocktail, Roche)). Homogenates were centrifuged at approximately 800 x g and 4 °C for 1 minute. The supernatants were collected, vortexed, aliquoted, snap-frozen in liquid nitrogen, and stored at -80 °C. Right before use, the substrate was thawed in ice and supplemented with 5 mM EDTA (Promega) and digitonin 0,025% (Invitrogen).

**PMCA reactions on swabs and tissues.** Ten µL of the 20% w/v tongue homogenates, or approximately 0.0025 g piece of the swab cotton tips were directly mixed with 90 µL of PMCA substrate. Cotton tips were cut in clean disposable containers, using new and disposable blades. The PMCA procedure was performed as extensively described in our previous publications (1–3). Briefly, a first PMCA round was conducted by submitting sample/substrate mixtures to 144 cycles of incubation and sonication (each PMCA cycle included 29 min and 40 s of incubation, and 20 s of sonication). The resulting materials were subjected to 3 additional rounds of PMCA (96 cycles each) by mixing 10 µL of the PMCA products of each round with new PMCA substrate (90 µL). PMCA products were treated with proteinase K (PK) and examined by western blotting. As controls, each PMCA reaction set (testing approximately 10 samples) included serial dilutions of a CWD brain of known PMCA activity and at least four unseeded reactions (NC).

**Proteinase K (PK) treatments.** To assess the potential presence of disease-associated prion proteins (PrP^Sc^), 20 µL of PMCA products or tongue homogenates were treated with 100 µg/mL PK (Sigma-Aldrich) at 37 °C and 450 rpm shaking for 60 min using and Eppendorf thermomixer. PK reactions were stopped by adding LDS sample buffer 1X (NuPAGE™) and exposure to 90 °C for 10 min. The resulting materials were examined by western blotting.

**Electrophoresis and western blotting.** Electrophoresis was performed in NuPAGE 4-12% or 12% Bis-Tris gels (Invitrogen) using MOPS Buffer (NuPAGE™) at 80V for 20 min, and then 140V for 1h 40 min. Fractionated proteins were transferred to nitrocellulose membranes (Cytiva Amersham) at 100 V and 4 °C for 60 min. Membranes were blocked using 10% (w/v) non-fat milk (Lab Scientific bioKEMIX) and probed with the primary monoclonal 8H4 antibody (Sigma-Aldrich) diluted at 1:5,000. A secondary polyclonal Anti-Mouse IgG (whole molecule)–Peroxidase antibody produced in sheep (Sigma-Aldrich) diluted at 1:3,000 was then used on the same membranes. Both primary and secondary antibodies were prepared in PBST- 0,05% and incubated for 1 h at room temperature. Membranes were washed three times for 10 min with PBST - 0,05% after being incubated with each antibody. Following provider instructions, the membrane was developed using ECL (Cytiva Amersham) in a BioRad dark room.

**RT-QuIC.** To visualize the presence of prion seeding activity in tissue homogenates, 5 µL of 0.00002% w/v homogenate of the tongue tissues were added to 95 µL of RT-QuIC buffer containing PBS (Hyclone PBS, GE Healthcare Life Sciences), 350 mM NaCl (Sigma-Adrich), 1 mM EDTA (Promega), 0.002% SDS (Sigma-Adrich), 0.05X N2 supplement media (Gibco), 100 mM NaI (Sigma-Adrich), 0.01 mM ThT (Sigma-Adrich) and 0.1 mg/mL of recombinant PrP. As negative control, 5 µL of a 0.002% w/v retropharyngeal lymph node homogenate from a CWD non-detect white-tailed deer was included in each set of RT-QuIC reactions. As positive control, 5 µL of a 0.002% w/v retropharyngeal lymph node homogenate from a CWD positive white-tailed deer (as evaluated by IHC and PMCA) was included in each set of RT-QuIC reactions. RT-QuIC reactions were seeded in plate formats as previously described (4). The plates were sealed with sealing tape and placed on an OMEGA plate reader (BMG Labtech) at 48 °C using the following parameters: 1 min shaking at 700 rpm double orbital, 1 min resting, 448 nm excitation, 482 nm emission, bottom read, and gain set at 2100 arbitrary units. ThT fluorescence was measured every 15 minutes for a total time of 68 h. The amyloid formation ratio was calculated using the MARS software (BMG Labtech), and data were plotted on GraphPad as previously described (5). Samples were run in quadruplicates, and considered as positive if 3 out 4 replicates displayed seeding activity.

**REFERENCES**

1. Morales R, Duran-Aniotz C, Diaz-Espinoza R, Camacho M V, Soto C. 2012. Protein misfolding cyclic amplification of infectious prions. Nat Protoc 7:1397–1409.

2. Kramm C, Gomez-Gutierrez R, Soto C, Telling G, Nichols T, Morales R. 2019. In Vitro detection of Chronic Wasting Disease (CWD) prions in semen and reproductive tissues of white tailed deer bucks (Odocoileus virginianus). PLoS One 14:e0226560.

3. Kramm C, Pritzkow S, Lyon A, Nichols T, Morales R, Soto C. 2017. Detection of Prions in Blood of Cervids at the Asymptomatic Stage of Chronic Wasting Disease. Sci Rep 7:17241.

4. Davenport KA, Hoover CE, Denkers ND, Mathiason CK, Hoover EA. 2018. Modified protein misfolding cyclic amplification overcomes real-time quaking-induced conversion assay inhibitors in deer saliva to detect chronic wasting disease prions. J Clin Microbiol 56.

5. Henderson DM, Davenport KA, Haley NJ, Denkers ND, Mathiason CK, Hoover EA. 2015. Quantitative assessment of prion infectivity in tissues and body fluids by real-time quaking-induced conversion. J Gen Virol 96:210–219.
